# Development of shortened HIV-related stigma scales for young people living with HIV and young people affected by HIV in India

**DOI:** 10.1186/s12955-022-02030-9

**Published:** 2022-07-31

**Authors:** Ivan Marbaniang, Rohidas Borse, Shashikala Sangle, Aarti Kinikar, Amol Chavan, Smita Nimkar, Nishi Suryavanshi, Vidya Mave

**Affiliations:** 1 Byramjee-Jeejeebhoy Government Medical College - Johns Hopkins University Clinical Research Site, Pune, India; 2 Department of Epidemiology, McGill University, 2001 McGill College, Suite 1200, Montreal, QC H3A 1G1 Canada; 3 Department of Medicine, Byramjee-Jeejeebhoy Government Medical College, Pune, India; 4 Department of Paediatrics, Byramjee-Jeejeebhoy Government Medical College, Pune, India; 5 Johns Hopkins University School of Medicine, Baltimore, MD USA

**Keywords:** HIV, Stigma, Instrument, Short-form, Psychometrics, Young people, India

## Abstract

**Background:**

HIV-related stigma is associated with poor quality of life and poor healthcare-seeking behaviours in young people living with HIV (YPLHIV) and young people affected by HIV (YPAHIV). India has an estimated 120,000 YPLHIV and 4 million YPAHIV, but efforts to measure HIV-related stigma in them are sparse, impeded by the lack of measuring instruments. Here, we describe the development of the Pune HIV-Stigma Scale (PHSS) and modified-PHSS to measure HIV-related stigma among YPLHIV and YPAHIV, respectively, in India.

**Methods:**

We used data from a mental health study for YPLHIV and YPAHIV aged 15–25 years, conducted at Byramjee Jeejeebhoy Government Medical College & Sassoon General Hospitals, Pune, India, between August 2018 and June 2021. Findings from multiple confirmatory factor analyses and cognitive interviews guided the development of the 12-item PHSS. The modified-PHSS was developed by confirming the structure of the PHSS for YPAHIV. Convergent validity with Center for Epidemiological Studies Depression (CES-D) and UCLA Loneliness scales was assessed using Spearman’s correlation coefficients.

**Results:**

Model fit indices were good for both the PHSS (χ^2^ = 65.0, df = 48, *p* value: 0.052; root mean square error of approximation (RMSEA): 0.054; comparative fit index (CLI): 0.980; Tucker–Lewis index (TLI): 0.972; and standardized root mean square residual (SRMR): 0.067), and the modified-PHSS (χ^2^ = 56.9, df = 48, *p* value: 0.176; RMSEA: 0.045; CLI: 0.983; TFI: 0.976, and SRMR: 0.078). Spearman’s correlation coefficients indicated low to moderate convergent validity (ρ: 0.03–0.52) across different subscales of the PHSS and modified-PHSS. Cronbach’s alpha for the PHSS was 0.82 and for the modified-PHSS 0.81.

**Conclusion:**

We developed the first scales to measure HIV-related stigma among YPLHIV and YPAHIV in India. These concise scales can facilitate measurement of HIV-related stigma more frequently in research studies. We recommend that they be tested in different Indian languages.

**Supplementary Information:**

The online version contains supplementary material available at 10.1186/s12955-022-02030-9.

## Introduction

Young people living with HIV (YPLHIV) are more vulnerable to HIV-related stigma, compared to their adult counterparts [1, 2]. Several factors contribute to this increased vulnerability including their unique developmental phase marked by rapid physical and psychosocial transitions [2], social and economic marginalization due to their HIV status [2, 3], and the general lack of YPLHIV-friendly services to help them navigate through HIV-related challenges [2, 4]. This is concerning because HIV-related stigma is associated with high-risk sexual and substance use behaviors [5, 6], reduced adherence to HIV medication and disengagement from care [5, 7–10], often mediated by poor psychological health and maladaptive responses [2, 5, 11, 12].

Outside sub-Saharan Africa, India has the highest number of YPLHIV [13, 14], that account for 35% of the total HIV cases in the country [15]. Despite the persistence of discriminatory attitudes towards PLHIV in India [16], only a few studies have measured HIV-related stigma in the country [17, 18], and none of them have done so exclusively among YPLHIV. This makes the planning of stigma mitigating interventions among Indian YPLHIV challenging.

HIV-related stigma is a multi-faceted construct that encompasses internal traumatizing aspects of living with the disease, and external socio-cultural aspects related to moral valuations ascribed to HIV [12, 19]. Historically, operationalizing a definition for the quantification of HIV-related stigma posed difficulties and attempts at measuring it remained atheoretical, i.e., without distinguishing between different mechanisms of HIV-related stigma [19]. The HIV Stigma Framework proposed by Earnshaw and Chaudoir improved on atheoretical models [20], by specifying three distinct HIV-related stigma mechanisms experienced by PLHIV. These are (a) enacted stigma, or experiences of discrimination, stereotyping and/or prejudice from others; (b) anticipated stigma, or expectations of enacted stigma; and (c) internalised stigma, or the internalisation of negative feelings and beliefs about HIV by PLHIV. Each of these mechanisms is theorized to have differential impacts on the psychological, social, and physical components of health and well-being of PLHIV [19, 21].

The 40-item HIV stigma scale (HSS) developed by Berger and colleagues [22] distinguishes between the three HIV-related stigma mechanisms described by Earnshaw and Chaudoir in a single instrument, generating domain specific scores (i.e., scores related to the mechanisms) but also an overall score. It remains one of the most widely used instruments to measure HIV-related stigma globally [23]. A major challenge with administering the HSS is its cumbersome length [24], contributing to participant fatigue in research studies with extensive surveys [25], and making it impractical to use in high burden HIV clinics.

Many shorter modifications of the HSS exist for adults and YPLHIV. In India, Jeyaseelan et al. adapted it to a 25-item questionnaire for Tamil (a south Indian language) in a study sample with a mean age of 34 years [24]. Similarly, 10-item and 12-item scales for African American [26] and Thai YPLHIV [27], have been developed using the HSS. The construction of HIV-related stigma is both age-group specific and contextual to the socio-cultural milieu [2, 12, 28, 29]. Therefore, shorter HIV-related stigma scales developed for older Indian PLHIV or YPLHIV in different socio-cultural environments cannot be assumed to be valid representations of the experiences or perceptions of HIV-related stigma in Indian YPLHIV, unless tested.

Young people affected by HIV (YPAHIV) are the children of parents that are/were living with HIV [30]. Although HIV uninfected themselves, YPAHIV often experience HIV-related stigma by association i.e., as a secondary target in proximity to their parent(s) [31]. In a recent scoping review of 26 articles, the underlying mechanisms by which YPAHIV experience HIV-related stigma, and their associations with poor mental health were reported to be similar to those for YPLHIV [31]. The HSS has been previously adapted to measure HIV-related stigma among YPAHIV in the United States [32] and South Africa [33], but no adaptations currently exist for Indian YPAHIV.

In this manuscript, our primary aim is to develop a shortened HIV-related stigma scale that is culturally relevant for Indian YPLHIV, based on the HSS and its previous shortened iterations. Our secondary aim is to test the validity of a separate scale for YPAHIV, based on the structure of the shortened HIV-related stigma scale identified in our primary aim.

## Methods

### Study population and procedures

Data for analyses were obtained from a mental health study of YPLHIV and YPAHIV. Participants were recruited from the antiretroviral therapy (ART) center affiliated to Byramjee Jeejeebhoy Government Medical College and Sassoon General Hospitals (BJGMC & SGH), a publicly funded tertiary health care center in Pune, a city located in the state of Maharashtra, western India. In 2019, the state had the highest number of PLHIV (n = 396,000) in India [34]. The ART center caters to approximately 350 YPLHIV. Recruitments were done between August 2018 and June 2021.

Two study counsellors approached all YPLHIV between 15 and 25 years of age attending the ART center for HIV care. To enroll YPAHIV, PLHIV attending the ART center with children between 15 and 25 years of age were approached. To prevent accidental disclosure, only YPLHIV and YPAHIV aware of their own or their parent’s HIV status, respectively, were enrolled. Written informed assent and parental/guardian consent were required for participants < 18 years of age or informed consent for particiapants ≥ 18 years. Institutionalized young people were excluded from the study. All participants received a gift coupon worth 150 Indian rupees (approximately US$ 2).

All study scales were self-administered on handheld devices. Marathi (the locally spoken language) was used in all study proceedings, including study scales. Participants were first required to undergo a reading/comprehension test, using a paragraph from an eighth grade Marathi textbook used in publicly funded schools (In India, the average age to reach eighth grade literacy is between 12 and 14 years). Participants were excluded (YPLHIV: n = 6, YPAHIV: n = 2) if they were unable to read/comprehend the paragraph. After successfully completing the test, participants were provided handheld devices to complete the study scales. Study counselors were present in the study room if participants required any scale items to be explained or clarified, but study responses were hidden from them.

The Ethics Committee of Byramjee Jeejeebhoy Government Medical College and the Johns Hopkins University Institutional Review Board approved this study.

### Study measures

The HSS was used to assess HIV-related stigma among YPLHIV and YPAHIV. The HSS is scored on a 4-point Likert scale (1-strongly agree to 4-strongly disagree). Total scores range between 40 and 160, with higher scores indicating greater HIV-related stigma. The scale is further divided into four subscales, with each subscale having a different number of items. Items can load on to more than one subscale. The subscales measure: personalized stigma (18 questions, score range: 18–72); disclosure concerns (10 questions, score range: 10–40); concern about public attitudes (13 questions, score range: 13–52); and negative self-image (20 questions, score range: 20–80).

The Center for Epidemiological Studies Depression (CES-D) scale was used to assess depressive symptoms in those ≥ 18 years of age, and its modification, the Center for Epidemiological Studies Depression Scale for Children (CES-DC) for those < 18 years [35, 36]. Both scales are worded similarly and ask participants to rate how often they experienced depressive symptoms over the past week, using 20 items scored on a 4-point Likert scale (0—rarely or none of the time to 3—most or all the time). Total scores range between 0 and 60, with higher scores indicating greater depressive symptoms. The scales had good internal consistency for YPLHIV (Cronbach’s α: 0.86 for CED-D and 0.88 for CES-DC) and YPAHIV (Cronbach’s α: 0.91 CES-DC and 0.80 for CES-DC).

The UCLA Loneliness Scale version 3 was used to measure participants’ subjective feelings of loneliness and social isolation, using 20 questions scored on a 4-point Likert scale (1—Never to 4—Often). Total scores range between 20 and 80, with higher scores indicating greater perceived loneliness and social isolation [37]. The scale had good internal consistency for YPLHIV and YPAHIV (Cronbach’s α: 0.85 and 0.79, respectively).

### Scale adaptation and modification for YPAHIV

The HSS was first translated from English to Marathi. The translated scale items were verified for consistency, cultural relevancy, and comprehensibility by a review committee. The review committee included three study counsellors (graduates in social work, each with ≥ 5 years of conducting quantitative or qualitative research), and two study investigators (SN & IM) trained in mental health, instrument development and psychometrics. The scale approved by the review committee was then back translated into English to assess for original item equivalence, by two individuals unrelated to the study and not familiar with the HSS, proficient in both Marathi and English. Following this, the translated scale was re-tested for participant comprehensibility, demographic and cultural relevancy using cognitive interviews with 33 YPLHIV.

The scale item, “People I care about stopped calling after learning I have HIV”, was modified as, “People close to me have stopped calling me on the telephone, coming to my house, after learning I have HIV,” following cognitive interviews in which participants expressed that ‘calling’ as stated in the original (HSS) scale item should be qualified better. Other scale items were translated as they appear in the HSS with no additional modifications.

For YPAHIV, the same procedures as described for YPLHIV were followed. However, HSS items were reworded to reflect the HIV status of the participant’s parents. For example, the question, “Telling people I have HIV is risky” was modified as, “Telling people my parents are/were living with HIV is risky”. Cognitive interviews were conducted with 20 YPAHIV. The question, “I feel guilty because my parents have/had HIV” was reworded as “I feel ashamed because my parents have/had HIV”, based on cognitive interview findings in which participants described feeling shame and not guilt to have/have had HIV positive parents.

Participants that took part in cognitive interviews were invited to be part of the study only after three months had passed. Similar procedures were followed to adapt the CES-D, CES-DC, and UCLA Loneliness Scales into Marathi.

### Statistical analyses

We identified eight published shortened adaptations of the HSS. Two of these adapted scales were in English [26, 38], three in Swedish [39–41], one in Spanish [42], one in Thai [27], and one in Tamil (linguistically unrelated to Marathi) [24]. The number of questions in these adapted scales range between 10 and 39.

Internal consistency was assessed using Cronbach’s alpha for the overall scale and the subscales. We tested the four-factor structure (modeled after the HSS subscales) for the eight adapted scales and the HSS among YPLHIV using confirmatory factor analysis (CFA). These are described as *primary models*. As responses in the HSS and adapted scales are ordinal, a weighted least squares estimator with a diagonal weight matrix and robust standard errors, and a mean- and variance-adjusted chi-square (χ^2^) statistic were used. The four factors i.e., personalized stigma, disclosure concerns, negative self-image and public attitudes concern were modelled as latent variables. Correlation between latent variables was allowed, but we did not allow inter-error correlation. Factor loadings between items and latent variables were standardized. Model fit was evaluated using χ^2^ test, Root Mean Square Error of Approximation (RMSEA), Tucker–Lewis Index (TLI), Comparative Fit Index (CFI) and the Standardized Root Mean Square Residual (SRMR). Good model fit was indicated by a χ^2^ associated *p* value > 0.05, RMSEA < 0.08, TLI and CFI ≥ 0.90 and SRMR < 0.08 [43].

*Secondary models* were constructed by (a) replacing a subscale(s) with low Cronbach’s alpha in models with good fit indices, with a subscale(s) from the eight primary adapted scales with a higher Cronbach’s alpha; (b) combining different subscales with the highest Cronbach’s alpha values from the eight adapted scales. CFA was performed on all secondary models.

The final *identified model* was chosen based on four criteria, (a) good model fit indices; (b) consistency of items with the original four factor structure loadings i.e., factors that loaded on to the latent variables in the abridged model were a subset of the factors that loaded on the corresponding latent variables in the HSS; (c) absolute magnitude of factor loadings > 0.40 [44]; (d) demographic and cultural relevance, as judged by findings from cognitive interviews. The final identified model was then tested on the dataset of YPAHIV using CFA.

We used Bonferroni-corrected Spearman’s correlation coefficients to assess for correlations between the HSS and the scale identified. Internal construct validity was evaluated using correlations between subscales, and convergent validity using correlations between the HSS, the identified scale and with CES-D(C) and UCLA Loneliness scales. We hypothesized that the subscales would be positively correlated with each other, and, with depressive and loneliness scores, as reported in previous studies [32, 41, 42]. All evaluations were conducted separately for YPLHIV and YPAHIV.

All analyses were performed in R version 4.1.2 and Stata 17.0.

## Results

### Description of the study populations

Overall, 124 YPLHIV and 93 YPAHIV were enrolled. The median age was comparable between both groups, 19 years (IQR: 15–25 years).

Among YLPHIV, 48% (n = 59) were female, 41% (n = 49) had > 12 years of education, 60% (n = 74) were employed, and 43% (n = 54) lived in makeshift houses. Perinatal transmission of HIV was the predominant (84%, n = 104) mode of HIV acquisition. Among YPAHIV, 56% (n = 52) were female, 60% (n = 53) had > 12 years of education, 37% (n = 34) were employed, and 44% (n = 41) lived in makeshift houses (Table 1).

**Table 1:** Characteristics of YPLHIV and YPAHIV participants

	YPLHIV n (%)	YPAHIV n (%)
Total N	124	93
Median age (years) (IQR)	19 (15–25)	19 (15–25)
Biologically female	59 (47.6)	52 (55.9)
> 12 years of education	49 (40.8)	53 (60.2)
Student at enrollment	76 (63.3)	60 (68.2)
Employed	74 (59.7)	34 (36.6)
Both parents alive	36 (29.0)	28 (30.1)
Live in makeshift houses	54 (43.5)	41 (44.1)
No toilet in the house	31 (25.0)	22 (23.7)
Good to excellent general health (self-assessed)	96 (77.4)	88 (94.6)
Any tobacco or alcohol use in the past year	9 (7.3)	15 (16.1)
Perinatally infected	104 (83.8)	–
Median CD4 counts (cells/mm^3^) (IQR)	516 (322–697)	–

*YPLHIV* young people living with HIV, *YPAHIV* young people affected by HIV, *IQR* 25th to 75th percentile interquartile range

### Internal consistency

Comparing across primary models, internal consistency for the overall scale and the four subscales was highest for the HSS (Table 2). Among the adapted scales, internal consistency for the overall scale, personalised stigma and concern about public attitudes subscales was highest for the Lindberg et al. [41] described scale (Cronbach’s α: 0.94, 0.94, 0.77, respectively). For the disclosure subscale, internal consistency was highest for the Reinius et al. [40] described scale (Cronbach’s α: 0.70). For the negative self-image subscale, internal consistency was highest for the Franke et al. [42] and Rongkavilit et al. [27] described scales (Cronbach’s α: 0.84 for both) (Table 2).

**Table 2:** Values for Cronbach’s alpha and confirmatory factor analysis fit indices for the dataset of YPLHIV in Pune, India

	Original scale (HSS)	Adapted scales							
	Berger [22]	Jeyaseelan [24]	Bunn [38]	Wright^a^ [26]	Rongkavilit^a^ [27]	Franke [42]	Wiklander^a^ [39]	Reinius [40]	Lindberg [41]
Number of scale items	40	25	32	10	12	21	12	12	39
Original language	English	Tamil	English	English	Thai	Spanish	Swedish	Swedish	Swedish
**Cronbach’s alpha**									
Overall scale	0.94	0.93	0.92	0.80	0.86	0.90	0.81	0.82	0.94
Personalized stigma	0.94	0.92	0.92	0.71	0.80	0.80	0.71	0.79	0.94
Disclosure	0.74	0.60	0.69	0.32	0.43	0.67	0.53	0.70	0.69
Negative self-image	0.85	0.81	0.76	0.73	0.84	0.84	0.75	0.73	0.79
Public attitudes concerns	0.92	0.65	0.72	0.50	0.59	0.71	0.68	0.60	0.77
**Model fit indices**									
* p* value for χ^2^	< 0.001	< 0.001	< 0.001	0.019	0.089	< 0.001	< 0.001	0.014	< 0.001
RMSEA	0.057	0.072	0.065	0.071	0.048	0.079	0.097	0.064	0.065
CFI	0.945	0.957	0.932	0.970	0.992	0.927	0.925	0.976	0.932
TLI	0.940	0.952	0.926	0.954	0.989	0.916	0.896	0.968	0.926
SRMR	0.090	0.082	0.099	0.065	0.052	0.095	0.085	0.072	0.099

*HSS* 40-item HIV-stigma scale, *χ*^*2*^ chi-square associated *p* value, *RMSEA* root mean square error of approximation, *CFI* comparative fit index, *TLI* Tucker–Lewis index, *SRMR* standardized root mean square residual

^a^Shortened scales for younger populations: Wright (16–25 years); Rongkavilit (16–25 years); Wiklander (8–18 years). For Wiklander et al., we used the scale that was described in Table 1 of their paper that had 12 questions and not the final version of their shortened scale with 8 questions. This was done to maintain the 4-component structure of the original scale

### Findings for primary models

Of the adapted scales, the best CFA model fit indices for YPLHIV were seen for the Rongkavilit et al. [27] described scale (χ^2^ = 61.7, df = 48, *p* value: 0.089; RMSEA: 0.048; SRMR: 0.052; CLI and TFI: 0.992 and 0.989, respectively) (Table 2). However, two factors on the disclosure, one factor on the concern about public attitudes, and one factor on the negative-self image subscales, were not original components on the corresponding HSS subscales (Additional file 1: Table S1). Moreover, one of the factors had a low loading on disclosure (magnitude: − 0.07) (Additional file 1: Table S2). Additionally, findings from cognitive interviews with YPLHIV indicated that their perceptions about disclosure concerns and negative self-image were inconsistent with those described by Rongakvilit et al. [27]. For example, disclosure concerns in Rongkavilit et al. [27] are predominantly associated with disclosure in hindsight (*I regret having told some people that I have HIV, People I cared about stopped calling after learning I have HIV*). However, disclosure concerns expressed by YLPHIV in our study population were related to prospective fears of being discovered to be living with HIV. This scale was therefore not considered appropriate for our study population.

The adapted scale by Reinius et al. [40] had the second-best CFA model fit indices for YLPHIV (χ^2^ = 72.0, df = 48, *p* value: 0.014, RMSEA: 0.064, SRMR: 0.072, CLI: 0.976 and TFI: 0.968, respectively), without having most of the limitations described for Rongkavilit et al. [27]. However, the scale had low internal consistency for the concern about public attitudes subscale (Cronbach’s α: 0.60) (Table 2).

### Findings for the identified model for YPLHIV

Multiple secondary models were tested (Additional file 1: Table S3). Our final identified model was a 12-item scale, formed using the Reinius et al. [40] described personalized stigma, disclosure, and negative self-image subscales and the Wiklander et al. [39] described concerns about public attitudes subscale. We refer to this scale henceforth as the *Pune HIV-stigma scale (PHSS)*.

Identification of the PHSS was based on the selection criteria specified earlier, namely, (a) good CFA model fit indices (χ^2^ = 65.0, df = 48, *p* value: 0.052; RMSEA: 0.054; SRMR: 0.067; CLI and TFI: 0.980 and 0.972, respectively) (Table 3); (b) subscale factors identified were a subset of the original HSS subscale factors (Additional file 1: Table S1); (c) all factor loadings > 0.40; (Table 3) (d) cultural and demographic relevancy. For example, in cognitive interviews, concern about public attitudes in YPLHIV were reflected more in statements associated with stronger sentiments (*Most people believe a person who has HIV is dirty*, *Most people think that a person with HIV is disgusting)* expressed in Wiklander et al. [39] than statements associated with weaker sentiments (*Most people are uncomfortable around someone with HIV*) expressed in Reinius et al. [40].

**Table 3:** Factor loadings and confirmatory factor analysis fit indices for the PHSS and modified-PHSS

	Factor loadings	
	PHSS	Modified-PHSS
	YPLHIV	YPAHIV
**Personalized stigma** (Reinius et al.) [40]		
28. Some people avoid touching me once they know I/my parents have HIV*	0.99	0.96
29. People I care about stopped calling me on the telephone, coming to my house after learning I/my parents have HIV	0.86	0.72
36. I have lost friends by telling them I/my parents have HIV	0.64	0.78
**Disclosure concerns** (Reinius et al.) [40]		
4. Telling someone I/my parents have HIV is risky	0.84	0.74
6. I work hard to keep my HIV/my parents HIV status a secret	0.74	0.81
17. I am very careful who I tell that I/my parents have HIV	0.65	0.71
**Negative self-image** (Reinius et al.) [40]		
2. I feel guilty because I have HIV/I feel ashamed because my parents have HIV	0.71	0.74
3. People's attitudes about HIV make me feel worse about myself	0.84	0.74
7. I feel I am not as good a person as others because I/my parents have HIV	0.69	0.43
**Public attitudes concerns** (Wiklander et al.) [39]		
10. Most people believe that a person who has HIV is dirty	0.72	0.80
14. Most people think that a person with HIV is disgusting	0.73	0.86
16. Most people with HIV are rejected when others find out	0.72	0.79

	Fit indices	
*p* value for χ^2^	0.052	0.176
RMSEA	0.054	0.045
CFI	0.980	0.983
TLI	0.972	0.976
SRMR	0.067	0.078

*PHSS* Pune HIV Stigma Scale, *YPLHIV* young people living with HIV, *YPAHIV* young people affected by HIV, *RMSEA* root mean square error of approximation, *CFI* comparative fit index, *TLI* Tucker–Lewis index, *SRMR* standardized root mean square residual

*The scale item, ‘Some people avoid touching me once they know I/my parents have HIV’ is meant to be read as ‘Some people avoid touching me once they know I have HIV’ for the PHSS i.e., for YPLHIV, and ‘Some people avoid touching me once they know my parents have HIV’ for the modified-PHSS, i.e., YPAHIV. This format is followed throughout the table wherever applicable

### Findings for the identified model for YPAHIV

We refer to the PHSS with modifications in phrasing for YPAHIV henceforth as the *modified-PHSS*. The modified-PHSS showed good CFA model fit indices (χ^2^ = 56.9, df = 48, *p* value: 0.176; RMSEA: 0.045; SRMR: 0.078; CLI: 0.983 and TFI: 0.976) (Table 3). Cognitive interview findings also indicated that the conceptualization of stigma in YPAHIV was consistent with that expressed by YPLHIV (Table 4).

**Table 4:** Comparison between the HSS, PHSS and modified-PHSS for YPLHIV and YPAHIV in Pune, India

	HSS					PHSS & modified-PHSS	PHSS			Modified-PHSS		
		YPLHIV		YPAHIV			YPLHIV			YPAHIV		
	Theoretical score range	Median score (IQR)	α	Median score (IQR)	α	Theoretical score range	Median score (IQR)	α	ρ	Median score (IQR)	α	ρ
Overall	40–160	87.5 (69–106)	0.94	71 (59–88)	0.94	12–48	28 (13–33)	0.82	0.89	23 (17–27)	0.81	0.89
Personalized stigma	18–72	36 (24–45)	0.94	25 (21–35)	0.93	3–12	5 (3–7)	0.79	0.89	3 (3–5)	0.71	0.72
Disclosure	10–40	27 (24–33)	0.74	25 (19–30)	0.80	3–12	9 (7–11)	0.70	0.76	8 (6–10)	0.73	0.84
Negative self-image	13–52	27 (21–32)	0.85	20 (17–25)	0.80	3–12	6 (4–9)	0.73	0.79	4 (3–6)	0.54	0.83
Public attitudes concerns	20–80	43 (33–53)	0.92	34 (27–44)	0.91	3–12	7 (5–9)	0.68	0.70	6 (4–9)	0.77	0.72

*HSS* 40-item HIV-stigma scale, *PHSS* Pune HIV Stigma Scale, *YPLHIV* young people living with HIV, *YPAHIV* young people affected by HIV, *α* Cronbach’s alpha, *ρ* Spearman’s corrected correlation coefficients between the HSS and 12-item scales

### Correlations between the HSS, PHSS and modified-PHSS

Correlations between the overall HSS, and both PHSS and modified-PHSS were 0.89. We observed strong correlations (> 0.7) [45] between the subscales (Table 4).

### Internal construct validity and convergent validity

Inter-subscale correlations were weaker for the PHSS and modified-PHSS (albeit mostly significant), compared to correlations seen for inter HSS subscales (Table 5).

**Table 5:** Correlations between subscales for HSS, PHSS and modified-PHSS

	HSS for YPLHIV				HSS for YPAHIV			
	Personalized stigma	Disclosure	Negative self-image	Public attitudes concerns	Personalized stigma	Disclosure	Negative self-image	Public attitudes concerns
Personalized stigma	1.00				1.00			
Disclosure	0.61*	1.00			0.63*	1.00		
Negative self-image	0.77*	0.62*	1.00		0.81*	0.69*	1.00	
Public attitudes concerns	0.94*	0.75*	0.78*	1.00	0.89*	0.74*	0.79*	1.00

PHSS					Modified-PHSS			
Personalized stigma	1.00				1.00			
Disclosure score	0.19	1.00			0.17	1.00		
Negative self-image	0.39*	0.35*	1.00		0.42*	0.35*	1.00	
Public attitudes concerns	0.48*	0.29*	0.35*	1.00	0.40*	0.43*	0.35*	1.00

*HSS* 40-item HIV-stigma scale, *PHSS* Pune HIV Stigma Scale, *YPLHIV* young people living with HIV; *YPAHIV* young people affected by HIV

*Statistically significant corrected correlations *p* < 0.05

We observed low to moderate correlation (ρ: 0.03–0.52) [45] between the PHSS, the modified-PHSS and the CES-D(C) and UCLA-Loneliness scales (Table 6). However, correlation magnitudes were largely consistent with those observed for the HSS.

**Table 6:** Correlations between scale items and CES-D(C) depression and UCLA loneliness scores

	HSS				PHSS		Modified-PHSS	
	YPLHIV		YPAHIV		YPLHIV		YPAHIV	
	CES-D(C) scale	UCLA loneliness scale	CES-D(C) scale	UCLA loneliness scale	CES-D(C) scale	UCLA loneliness scale	CES-D(C) scale	UCLA loneliness scale
Overall	0.47*	0.43*	0.22*	0.22*	0.42*	0.41*	0.16	0.13
Personalized stigma	0.49*	0.44*	0.17	0.25*	0.48*	0.52*	0.13	0.22*
Disclosure	0.20*	0.25*	0.15	0.03	0.03	0.07	0.08	− 0.07
Negative self-image	0.43*	0.44*	0.21*	0.34*	0.40*	0.39*	0.21*	0.31*
Public attitudes concerns	0.41*	0.38*	0.23*	0.23*	0.28*	0.21*	0.10	0.09

*HSS* 40-item HIV-stigma scale, *PHSS* Pune HIV stigma scale, *YPLHIV* young people living with HIV, *YPAHIV* young people affected by HIV

*Statistically significant corrected correlations *p* < 0.05

## Discussion

We adapted the 40-item HSS to a 12-item PHSS and a 12-item modified-PHSS to assess HIV-related stigma among Indian YPLHIV and YPAHIV, respectively. The PHSS and modified-PHSS demonstrated good model fit indices, acceptable internal consistency, and good correlations with the HSS. To our knowledge, these scales are the first HIV-related stigma scales described for Indian YPLHIV and YPAHIV.

Compared to the 25-item scale by Jeyaseelan et al. [24], also adapted in India for older PLHIV, the PHSS fit our YPLHIV data better, and given its shorter length would take lesser time to administer. Additionally, the PHSS had higher internal consistency for the disclosure subscale (Cronbach’s α: 0.70 vs. 0.60), and comparable internal consistency for the other three subscales. Another significant difference between the scales is that the 25-item scale has two items that load on disclosure (*I regret having told some people that I have HIV*) and negative self-image (*Some people act as though it’s my fault that I have HIV*), that are not components described for the corresponding HSS subscales. Our cognitive interviews also indicated that perspectives on disclosure expressed by YPLHIV in our study population diverged from those measured by the 25-item scale. Specifically, disclosure concerns in the 25-item scale incorporate aspects of anticipated stigma (*I worry that people who know I have HIV will tell others, I worry that people may judge me when they learn I have HIV)* and enacted stigma (*I regret having told some people that I have HIV).* On the other hand, disclosure concerns among YPLHIV in our study population were expressed entirely in relation to anticipated stigma. These findings highlight the importance of retesting measuring instruments for different age groups even in settings with cultural similarities.

We found that overall PHSS scores were significantly correlated to CES-D(C) and UCLA-Loneliness scores, and correlation strength was consistent with that observed for the HSS. However, of noteworthy difference was that correlations for the PHSS disclosure scores were non-significant. A primary reason for this could be that five of ten HSS disclosure items load multiply on other subscales. Therefore, significant correlations observed with HSS disclosure scores, could be driven by underlying correlations with other latent constructs.

Interestingly, relative to the significant positive correlations observed between overall HSS scores with CES-D(C) and UCLA-Loneliness scores, correlations with overall modified-PHSS scores and most of the modified-PHSS subscales were non-significant. We are unable to explain these observations completely. However, given the low correlational strength observed for YPAHIV even for the 40-item HSS, we posit two hypotheses: (a) HIV-related stigma may be less correlated with depressive symptoms and loneliness among YPAHIV than YPLHIV in India; (b) the 40-item HSS may not be suitable for adaptation to measure HIV-related stigma among Indian YPAHIV. Future studies should investigate better the experiences and perceptions of stigmatization in Indian YPAHIV, and their associations with depressive symptoms and loneliness. Additionally, given that there is limited research on HIV-related stigma among Indian YPAHIV, we hope that by making explicit the limitations of the modified-PHSS, we will encourage researchers to develop better HIV-related stigma measuring instruments for this population.

There are several limitations to our study that merit discussion. Given the small sample sizes for both YLPHIV and YPAHIV, we were unable to evaluate if the scales are invariant by gender. Stigma has gendered connotations [29, 46], and it is important to evaluate this in the future. Additionally, due to the cross-sectional nature of our study, we were unable to assess if the scales are temporally invariant. We recommend that they be further tested in longitudinal studies to better understand the temporal construction of HIV-related stigma [19]. Our small sample size also precluded dividing datasets into two halves, to first perform an exploratory factor analysis followed by CFA. However, for YPLHIV, using findings from multiple CFA and cognitive interviews together, allowed us to develop the PHSS which is informed by both robust statistical analyses and practical considerations. Moreover, the dataset for YPLHIV is one of the largest datasets globally [47], in which HIV-related stigma has been measured. We modelled the structure of the modified-PHSS after the PHSS, to facilitate a direct comparison of HIV-related stigma between YPLHIV and YPAHIV. As indicated by the poorer convergent validity of the modified-PHSS, this approach may be lacking and we advice the precautious use of this scale. We were also unable to evaluate test–retest reliability for the scales, as retesting planned in the latter half of the main study, was limited due to COVID-19 pandemic restrictions. Lastly, we are unable to comment on the validity of these scales to measure HIV-related stigma for YPLHIV and YPAHIV that occupy intersectional positions based on their sexual or gender identities, caste, and socio-economic class. Since HIV-related stigma may be higher and constructed differently among such individuals [12, 19], we advocate for more research on the subject.

The PHSS and modified-PHSS are two instruments that may enable researchers to measure HIV-related stigma quickly and more regularly among YPLHIV and YPAHIV in India. We recommend the testing of these scales in different geographical regions of India in larger studies, simultaneously with longitudinal evaluations, to corroborate their reliability and validity.

## Supplementary Information

**Additional file 1.** **Supplementary Table 1:** Original Berger scale items (HSS) and factors that load on subscales. **Supplementary Table 2:** Factor loadings for the scales described by Rongkavilit et al. and Reinius et al. for YPLHIV. **Supplementary Table 3:** Confirmatory factor analysis model fit indices for a combination of stigma scales from different authors for YPLHIV. **Supplementary Figure 1:** Path diagram for PHSS. **Supplementary Figure 2:** Path diagram for modified-PHSS.

## Data Availability

The datasets used for this manuscript are available from the corresponding author on reasonable request. Investigators interested in obtaining the PHSS or modified-PHSS in Marathi are requested to contact the corresponding author.

## Abbreviations

UNAIDS: United Nations Joint Program on HIV/AIDS
SDGs: Sustainable Development Goals
PLHIV: People living with HIV
YPLHIV: Young people living with HIV
HSS: 40-Item HIV stigma scale
YPAHIV: Young people affected by HIV
ART: Antiretroviral therapy
BJGMC & SGH: Byramjee Jeejeebhoy Government Medical College and Sassoon General Hospitals
CES-D: Center for Epidemiological Studies-Depression Scale
CES-DC: Center for Epidemiological Studies Depression Scale for Children
CFA: Confirmatory factor analysis
RMSEA: Root mean square error of approximation
TLI: Tucker–Lewis Index
CFI: Comparative Fit Index
SRMR: Standardized root mean square residual
PHSS: Pune HIV-stigma scale
