# Supplementary material for: Development of shortened HIV-related stigma scales for young people living with HIV and young people affected by HIV in India
Source: Health Qual Life Outcomes. 2022 Jul 31;20:119. doi: 10.1186/s12955-022-02030-9 (PMC9340676; doi:10.1186/s12955-022-02030-9)

| **Supplementary Table 1: Original Berger scale items and factors that load on subscales** | | | | | | | | | |
| --- | --- | --- | --- | --- | --- | --- | --- | --- | --- |
| The subscales present in the original 4-factor structure of the Berger questionnaire include: **Personalized stigma** (**PS**); **Disclosure (D); Negative self- image (NSI); Public attitudes (PA)** | | | | | | | | | |
|  | **Berger^a^** | **Jeyaseelan** | **Bunn** | **Wright** | **Rongkavilit** | **Franke** | **Wiklander** | **Reinius** | **Lindberg** |
| **Original 40-item Berger scale items** | | | | | | | | | |
| 1. In many areas of my life, no one knows that I have HIV | **D** |  | **D** |  | **D** |  |  |  | **D** |
| 1. I feel guilty because I have HIV | **NSI** |  | **NSI** |  |  | **NSI** |  | **NSI** | **NSI** |
| 1. People's attitudes about HIV make me feel worse about myself | **NSI** | **NSI** | **NSI** |  | **PA** |  |  | **NSI** | **NSI** |
| 1. Telling someone I have HIV is risky | **D, PA** |  | **D** |  |  | **D** |  | **D** | **D** |
| 1. People with HIV lose their jobs when their employers find out | **PA** |  | **PA** |  |  |  |  |  | **PA** |
| 1. I work hard to keep my HIV a secret | **D, NSI** |  | **D** |  |  | **D** | **D** | **D** | **D** |
| 1. I feel I am not as good a person as others because I have HIV | **NSI** | **NSI** | **NSI** | **NSI** |  | **NSI** |  | **NSI** | **NSI** |
| 1. I never feel ashamed of having HIV | **NSI** |  | **NSI** |  |  |  |  |  | **NSI** |
| 1. People with HIV are treated like outcasts | **PA** |  | **PA** |  | **PA** | **PA** |  | **PA** | **PA** |
| 1. Most people believe that a person who has HIV is dirty | **PA** |  | **PA** |  | **PA** | **PA** | **PA** | **PA** | **PA** |
| 1. It is easier to avoid new friendships than worry about telling someone that I have HIV | **D, NSI, PA** |  |  |  |  |  |  |  |  |
| 1. Having HIV makes me feel unclean | **NSI** | **NSI** | **NSI** | **NSI** |  | **NSI** | **NSI** |  | **NSI** |
| 1. Since learning I have HIV, I feel set apart and isolated from the rest of the world | **PS, NSI, PA** | **NSI** |  |  |  | **NSI** |  |  | **NSI** |
| 1. Most people think that a person with HIV is disgusting | **PA** |  | **PA** | **PA** |  | **PA** | **PA** |  | **PA** |
| 1. Having HIV makes me feel that I'm a bad person | **NSI** | **NSI** | **NSI** | **NSI** |  | **NSI** | **NSI** |  | **NSI** |
| 1. Most people with HIV are rejected when others find out | **PS, PA** |  | **PA** | **PA** |  | **PA** | **PA** |  | **PA** |
| 1. I am very careful who I tell that I have HIV | **D** |  | **D** | **D** |  | **D** | **D** | **D** | **D** |
| 1. Some people who know I have HIV have grown more distant | **PS** | **PS** | **PS** |  |  | **PS** |  |  | **PS** |
| 1. Since learning I have HIV, I worry about people discriminating against me | **D, PA** |  |  |  |  |  |  |  | **PA** |
| 1. Most people are uncomfortable around someone with HIV | **PA** |  | **PA** |  |  | **PA** |  | **PA** | **PA** |
| 1. I never feel the need to hide the fact that I have HIV | **D** | **D** | **D** |  |  |  |  |  | **D** |
| 1. I worry that people may judge me when they learn I have HIV | **D, PA** | **D** | **D** |  |  | **D** |  |  | **D** |
| 1. Having HIV in my body is disgusting to me | **NSI** |  | **NSI** |  |  | **NSI** | **NSI** |  | **NSI** |
| 1. I have been hurt by how people reacted to learning I have HIV | **PS** | **PS** | **PS** | **PS** |  |  | **PS** |  | **PS** |
| 1. I worry that people who know I have HIV will tell others | **D** | **D** | **D** | **D** |  | **D** | **D** |  | **D** |
| 1. I regret having told some people that I have HIV | **PS** | **D** | **PS** |  | **D** | **PS** |  |  | **PS** |
| 1. As a rule, telling others that I have HIV has been a mistake | **PS, NSI, PA** |  |  |  |  | **PS** |  |  | **PS** |
| 1. Some people avoid touching me once they know I have HIV | **PS, PA** | **PS** | **PS** |  |  |  |  | **PS** | **PS** |
| 1. People I care about stopped calling after learning I have HIV | **PS** | **PS** | **PS** |  | **D** |  |  | **PS** | **PS** |
| 1. People have told me that getting HIV is what I deserve for how I lived my life | **PS, PA** |  |  |  |  |  |  |  | **PS** |
| 1. Some people close to me are afraid others will reject them if it becomes known that I have HIV | **PS** | **PS** |  |  |  |  |  |  | **PS** |
| 1. People don't want me around their children once they know I have HIV | **PS, PA** | **PS** | **PS** |  |  |  |  |  | **PS** |
| 1. People have physically backed away from me when they learn I have HIV | **PS, PA** | **PS** | **PS** |  |  |  |  |  | **PS** |
| 1. Some people act as though it's my fault I have HIV | **PS, PA** | **NSI** |  |  | **PS** | **PS** |  |  | **PS** |
| 1. I have stopped socializing with some people because of their reactions to my having HIV | **PS** | **PS** | **PS** | **PS** | **PS** |  | **PS** |  | **PS** |
| 1. I have lost friends by telling them I have HIV | **PS** | **PS** | **PS** | **PS** | **PS** |  | **PS** | **PS** | **PS** |
| 1. I have told people close to me to keep the fact that I have HIV a secret | **D** |  | **D** |  |  |  |  |  | **D** |
| 1. People who know I have HIV tend to ignore my good points | **PS, NSI, PA** | **PS** | **PS** |  | **NSI** |  |  |  | **PS** |
| 1. People seem afraid of me once they learn I have HIV | **PS, NSI, PA** | **PS** | **PS** |  | **NSI** | **PS** |  |  | **PS** |
| 1. When people learn you have HIV, they look for flaws in your character | **PS, PA** |  |  |  | **NSI** |  |  |  | **PS** |
| ^a^ Factors cross-loaded on the original Berger subscales but not on the other scales | | | | | | | | | |

| **Supplementary Table 2: Factor loadings for the scales described by Rongkavilit and Reinius for YPLHIV** | |
| --- | --- |
| **Scale: Rongkavilit et al.** | **Factor loadings** |
| **Personalized stigma**  34. Some people act as though it’s my fault I have HIV  35. I have stopped socializing with some people because of their reactions to my having HIV  36. I have lost friends by telling them I have HIV |  |
|  | 0.92 |
|  | 0.82 |
|  | 0.77 |
| **Disclosure concerns**  1. In many areas of my life, no one knows I have HIV  26. I regret having told some people that I have HIV  29. People I cared about stopped calling after learning have HIV |  |
|  | -0.01 |
|  | 0.72 |
|  | 0.90 |
| **Negative self-image**  38. People who know I have HIV tend to ignore my good points  39. People seem afraid of me once they learn I have HIV  40. When people learn you have HIV they look for flaws in your character |  |
|  | 0.83 |
|  | 0.92 |
|  | 0.86 |
| **Public attitudes concern**  3. People’s attitudes make me feel worse about myself  9. People with HIV are treated like outcasts  10. Most people believe that a person who has HIV is dirty |  |
|  | 0.57 |
|  | 0.73 |
|  | 0.61 |
|  | |
| **Scale: Reinius et al.** | **Factor loadings** |
| **Personalized stigma**  28. Some people avoid touching me once they know I have HIV  29. People I care about stopped calling after learning I have HIV  36. I have lost friends by telling them I have HIV |  |
|  | 0.92 |
|  | 0.93 |
|  | 0.67 |
| **Disclosure concerns**  4. Telling someone I have HIV is risky  6. I work hard to keep my HIV status a secret  17. I am very careful who I tell that I have HIV |  |
|  | 0.84 |
|  | 0.74 |
|  | 0.64 |
| **Negative self-image**  2. I feel guilty because I have HIV  3. People's attitudes about HIV make me feel worse about myself  7. I feel I am not as good a person as others because I have HIV |  |
|  | 0.76 |
|  | 0.78 |
|  | 0.75 |
| **Public attitudes concern**  9. People with HIV are treated like outcasts  10. Most people believe a person who has HIV is dirty  20. Most people are uncomfortable around someone with HIV |  |
|  | 0.61 |
|  | 0.56 |
|  | 0.75 |
|  | |

| **Supplementary Table 3: Confirmatory factor analysis model fit indices for a combination of stigma scales from different authors** | | | | | | | | | |
| --- | --- | --- | --- | --- | --- | --- | --- | --- | --- |
|  | **Reinius**  **(PS, D, NSI)**  **+**  **Wiklander**  **(PA)** | **Jeyaseelan (PS)**  **+**  **Reinius**  **(D)**  **+**  **Franke**  **(NSI)**  **+**  **Lindberg**  **(PA)** | **Rongkavilit**  **(PS, NSI)**  **+**  **Reinius**  **(D)**  **+**  **Lindberg**  **(PA)** | **Rongkavilit**  **(PS)**  **+**  **Reinius**  **(D)**  **+**  **Bunn**  **(NSI)**  **+**  **Lindberg**  **(PA)** | **Rongkavilit**  **(PS, NSI, PA)**  **+**  **Reinius**  **(D)** | **Rongkavilit**  **removing low loading**  **factor *** | **Reinius**  **(PS, D, NSI)**  **+**  **Lindberg**  **(PA)** | **Reinius**  **(PS, D, NSI)**  **+**  **Bunn**  **(PA)** | **Reinius**  **(PS, D, NSI)**  **+**  **Franke**  **(PA)** |
| **Reason for combination** | **Best model fit indices but replacing the subscale with the lowest internal consistency** | Subscales with the highest  Cronbach’s alpha | Subscales with the highest  Cronbach’s alpha | Subscales with the highest  Cronbach’s alpha | Best model fit indices but replacing the subscale for disclosure (Rongkavilit’s disclosure subscale has low internal consistency) | Best model fit indices but removing the low loading item | Best model fit indices but replacing the subscale with lowest internal consistency | Best model fit indices but replacing the subscale with lowest internal consistency | Best model fit indices but replacing the subscale with lowest internal consistency |
| **No of items** | **12** | 27 | 16 | 20 | 12 | 11 | 16 | 15 | 14 |
| **Model fit indices** |  |  |  |  |  |  |  |  |  |
| p-value for χ2 | 0.052 | p<0.001 | 0.001 | <0.001 | 0.120 | 0.044 | <0.001 | <0.001 | 0.001 |
| RMSEA | 0.054 | 0.060 | 0.065 | 0.073 | 0.044 | 0.059 | 0.075 | 0.076 | 0.068 |
| CFI | 0.980 | 0.963 | 0.972 | 0.928 | 0.991 | 0.990 | 0.952 | 0.950 | 0.964 |
| TLI | 0.972 | 0.959 | 0.966 | 0.917 | 0.987 | 0.986 | 0.941 | 0.937 | 0.954 |
| SRMR | 0.067 | 0.081 | 0.074 | 0.094 | 0.058 | 0.048 | 0.083 | 0.076 | 0.077 |
| PS- Personalized stigma scale; D -Disclosure scale; NSI- negative self-image scale; PA – Public attitudes concerns scale  *Question 1 of the original Berger stigma scale had a factor loading of negative 0.007 on the latent variable for disclosure which was excluded | | | | | | | | | |

| **Supplementary Table 3 (continued): Confirmatory factor analysis model fit indices for a combination of stigma scales from different authors** | | | | | | | | |
| --- | --- | --- | --- | --- | --- | --- | --- | --- |
|  | **Bunn**  **(PS, D)**  **+**  **Franke**  **(NSI)**  **+**  **Bunn**  **(PA)** | **Bunn**  **(PS)**  **+**  **Lindberg**  **(D)**  **+**  **Jeyaseelan**  **(NSI)**  **+**  **Franke**  **(PA)** | **Lindberg**  **(PS)**  **+**  **Reinius**  **(D)**  **+**  **Franke**  **(NSI)**  **+**  **Lindberg**  **(PA)** | **Lindberg**  **(PS, D)**  **+**  **Jeyaseelan**  **(NSI)**  **+**  **Franke**  **(PA)** | **Jeyaseelan**  **(PS)**  **+**  **Lindberg**  **(D)**  **+**  **Rongkavilit**  **(NSI)**  **+**  **Bunn**  **(PA)** | **Jeyaseelan**  **(PS)**  **+**  **Bunn**  **(D)**  **+**  **Jeyaseelan**  **(NSI)**  **+**  **Franke**  **(PA)** | **Bunn (PS)**  **+**  **Reinius (D)**  **+**  **Rongkavilit (NSI)**  **+**  **Lindberg**  **(PA)** | **Rongkavilit**  **(PS)**  **+**  **Reinius**  **(D)**  **+**  **Rongkavilit**  **(NSI)**  **+**  **Lindberg**  **(PA)** |
| **Reason for combination** | Subscales with the highest  Cronbach’s alpha | Subscales with the highest  Cronbach’s alpha | Subscales with the highest  Cronbach’s alpha | Subscales with the highest  Cronbach’s alpha | Subscales with the highest  Cronbach’s alpha | Subscales with the highest  Cronbach’s alpha | Subscales with the highest  Cronbach’s alpha | Subscales with the highest  Cronbach’s alpha |
| **No of items** | 32 | 30 | 32 | 35 | 26 | 31 | 22 | 16 |
| **Model fit indices** |  |  |  |  |  |  |  |  |
| p-value for χ2 | <0.001 | <0.001 | <0.001 | <0.001 | <0.001 | <0.001 | <0.001 | <0.001 |
| RMSEA | 0.063 | 0.071 | 0.056 | 0.063 | 0.072 | 0.071 | 0.063 | 0.065 |
| CFI | 0.944 | 0.939 | 0.964 | 0.926 | 0.948 | 0.941 | 0.972 | 0.972 |
| TLI | 0.939 | 0.939 | 0.961 | 0.920 | 0.942 | 0.936 | 0.968 | 0.966 |
| SRMR | 0.094 | 0.098 | 0.080 | 0.096 | 0.092 | 0.097 | 0.075 | 0.074 |
| PS- Personalized stigma scale; D -Disclosure scale; NSI- negative self-image scale; PA – Public attitudes concerns scale | | | | | | | | |

**Supplementary Figure 1**: Path diagram for PHSS


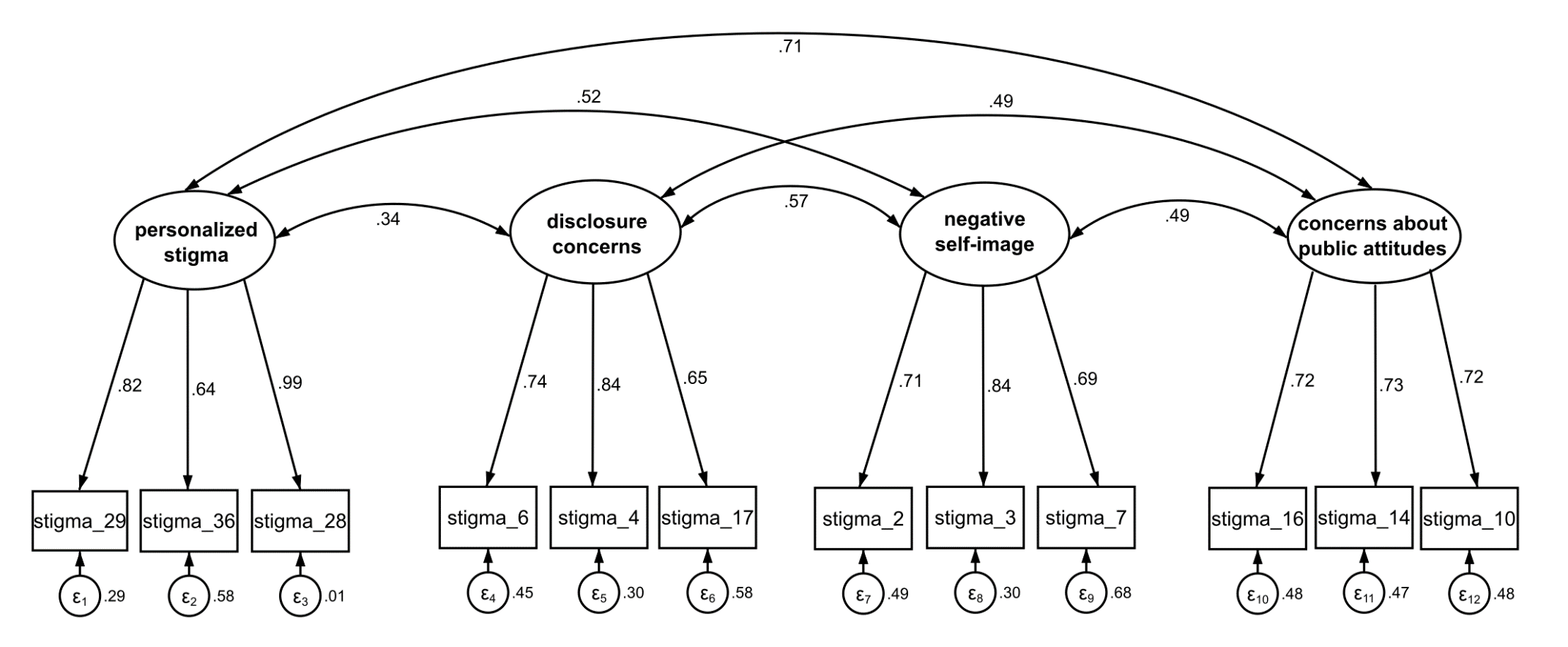


**Supplementary Figure 2**: Path diagram for modified-PHSS


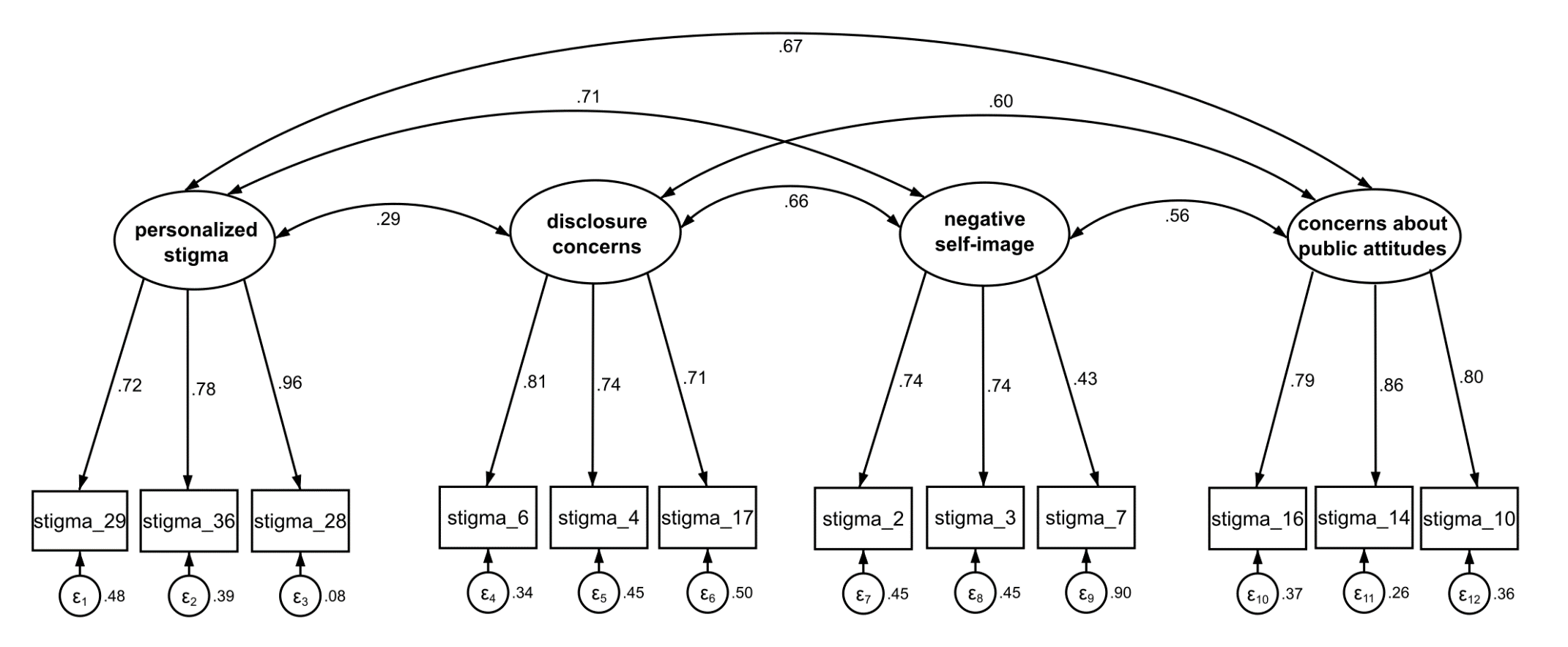

Supplement: Supplementary file 1 — Additional file 1. Supplementary Table 1: Original Berger scale items (HSS) and factors that load on subscales. Supplementary Table 2: Factor loadings for the scales described by Rongkavilit et al. and Reinius et al. for YPLHIV. Supplementary Table 3: Confirmatory factor analysis model fit indices for a combination of stigma scales from different authors for YPLHIV. Supplementary Figure 1: Path diagram for PHSS. Supplementary Figure 2: Path diagram for modified-PHSS. [file 12955_2022_2030_MOESM1_ESM.docx]
